# Current and Future Advancements of Raman Spectroscopy Techniques in Cancer Nanomedicine

**DOI:** 10.3390/ijms222313141

**Published:** 2021-12-05

**Authors:** Elisabetta Canetta

**Affiliations:** Faculty of Sport, Applied Health and Performance Science, St Mary’s University, Twickenham, London TW1 4SX, UK; elisabetta.canetta@stmarys.ac.uk

**Keywords:** cancer, nanomedicine, extracellular vesicles, Raman scattering, surface-enhanced Raman scattering, spatially offset Raman spectroscopy, nanotheranostics, SERS immunoassay, immuno-SERS microscopy

## Abstract

Raman scattering is one of the most used spectroscopy and imaging techniques in cancer nanomedicine due to its high spatial resolution, high chemical specificity, and multiplexity modalities. The flexibility of Raman techniques has led, in the past few years, to the rapid development of Raman spectroscopy and imaging for nanodiagnostics, nanotherapy, and nanotheranostics. This review focuses on the applications of spontaneous Raman spectroscopy and bioimaging to cancer nanotheranostics and their coupling to a variety of diagnostic/therapy methods to create nanoparticle-free theranostic systems for cancer diagnostics and therapy. Recent implementations of confocal Raman spectroscopy that led to the development of platforms for monitoring the therapeutic effects of anticancer drugs in vitro and in vivo are also reviewed. Another Raman technique that is largely employed in cancer nanomedicine, due to its ability to enhance the Raman signal, is surface-enhanced Raman spectroscopy (SERS). This review also explores the applications of the different types of SERS, such as SERRS and SORS, to cancer diagnosis through SERS nanoprobes and the detection of small-size biomarkers, such as exosomes. SERS cancer immunotherapy and immuno-SERS (iSERS) microscopy are reviewed.

## 1. Introduction

Cancer is one of the most common diseases that burden our society and creates stressful situations for individuals and their families from physical, emotional, and financial viewpoints. According to the WHO (World Health Organization), cancer is the second global leading cause of death and different types of cancer affect men and women dissimilarly; for example, stomach and liver cancers are most common in men, whilst thyroid and breast are most frequent in women [1]. As estimated by GLOBOCAN 2020, the burden generated by cancer globally is expected to reach 28.4 million cases in 2040, which equates to a 47% increase from the number of cases recorded in 2020 [2]. 

A key to the successful treatment of cancer is early detection [3,4] because of the substantial decrease in mortality that the detection of tumoral lesions and masses in the early stages of the illness can produce [5,6,7]. At the moment, early diagnosis of cancer is achieved by using a range of imaging techniques, such as Computed Tomography (CT), blue laser endoscopy, Magnetic Resonance Imaging (MRI), fluorescence molecular imaging [8,9,10,11,12], histopathology [13,14,15], and cytology [16,17,18]. Interestingly, when histopathology and cytology are employed as probing tools for determining the level of malignancy of an early-stage tumour [19], they are not used alone but in conjunction with standard imaging techniques (e.g., CT, MRI, Positron Emission Tomography (PET), and ultrasounds). Furthermore, the latter does not have the capabilities to provide the molecular information that would be clinically necessary to accurately identify the different types of cancers and their stages [20]. The necessity to improve the accuracy and reliability of early diagnosis of cancer has led to a rapid increase not only in investigating the clinical use of nanotechnology tools, techniques, and systems for cancer screening [21] but also in using nanomaterials as biosensors for the detection of cancer biomarkers in bodily fluids [22]. The benefit of using nanotechnology for cancer diagnosis lies in the high complexity and multifactorial nature of the disease that originate from cellular abnormalities in genetic and molecular processes [23]. 

Effective cancer imaging and screening processes have proved to be of paramount importance in the early detection of cancer, and the last decade has seen rapid advancements in the development and implementation of the efficacy and sensitivity of contrast agents, such as fluorescent and plasmonic (e.g., gold, silver, and copper) nanoparticles [24]. Furthermore, cancer nanomedicine has made expeditious progress in cancer diagnosis and therapy. 

Nanomedicine is a relatively new field of research that has emerged at the end of the 20th century [25] and is rapidly becoming a pivotal technology in 21st century medicine [26,27,28]. It began to play a key role in cancer therapy when the enhanced permeability and retention (EPR) effect [29] was enhanced by overcoming the various obstacles encountered in the delivery of nanodrugs to cancer sites [30]. The EPR effect occurs when nanoscale agents exploit the permeable cancer vasculature to accumulate into solid tumours and they remain in the cancer site because of reduced lymphatic drainage. Cancer nanomedicine exploits the EPR effect to increase the number of nanodrugs delivered to the cancer site and, therefore, the accumulation of anticancer therapeutics in tumour tissues [31]. However, one of the challenges when designing anticancer drug delivery systems is the select targeting of all cancer cells while reducing nanotoxicity effects on healthy tissues. Nanoparticles play a pivotal role in drug delivery because they not only act as nanocarriers of the antitumor drug but also allow for a very accurate release of the drug to the cancer site [32]. Another advantage of nanocarrier systems for cancer targeting is that they are designed to overcome the biological barriers (e.g., the immune and renal systems, and the blood–brain barrier) that the drugs would otherwise encounter when travelling towards the tumour [33]. Nanocarriers can be tailored to utilize:

(1) Passive targeting mechanisms by exploiting the EPR effect;
(2) Active targeting methods by employing ligands attached onto the surface of the nanocarriers as highly specific receptors that link only to one type of cancer cell [34].

However, the efficacy of using the EPR effect for passive tumour targeting has been recently questioned because the heterogeneity of the tumour vasculature introduces variability in the accumulation of nanomaterials in solid cancers, making the EPR effect highly influenced by the characteristics of the tumour microenvironment [35]. 

To overcome the issues related to the classical EPR effect and ensure the effectiveness of cancer therapy, nanotheranostics approaches have been developed. Theranostics is defined as the combination of diagnostic tests and ad hoc therapy when the latter is informed by test results [36]. Cancer nanotheranostics benefits greatly from the design, implementation, and synthesis of new nanoparticles, and their ability to deliver multiple drugs to the tumours due to the subsequent optimization of cancer therapies [37]. The novel nanotherapeutic modalities offered by nanotheranostics have been used in nanoparticle-assisted cancer immunotherapy [38], where nanoparticles are used as nanocarriers for the delivery of vaccines, cytokines, and antagonistic antibodies. An example is the use of nanomaterial-based vaccination strategies for further implementing T-cell cancer immunotherapy by inducing T-cell activation in vivo [39]. Another advantage of nanomaterial-assisted cancer immunotherapy is that by employing nanoparticles as delivery systems, the toxicity usually associated with immunotherapy can be avoided [40]. Despite the rapid progress in the development and implementation of increasingly sophisticated and multifunctional cancer nanotherapeutics, only a few of these nanodrugs are translated into clinically approved anticancer drugs [41]. Nanotheranostics is also employed for designing hybrid smart nanoplatforms where functional nanoparticles are used in the therapeutic modality for the release of chemotherapeutic drugs in response to an external stimulus [42]. Peng et al. [43] developed a nanotheranostic platform where epigallocatechin gallate (PEG), which can induce apoptosis in prostate cancer cells by inhibiting matrix metalloproteinases, was encapsulated into fucoidan/hyaluronic acid/PEG-gelatin (FU/HA/PG)-coated Poly (D,L-Lactide-co-Glycolide) (PLGA) nanoparticles. The efficacy of the nanotheranostic platform was tested in an orthotopic prostate cancer mouse model. FU/HA/PG-coated–PLGA-encapsulated iron oxide nanoparticles were used to obtain in vivo molecular imaging of the prostate tumour so that the diagnostic modality of the smart platform could be tested. The metabolic and physiological characteristics of the prostate cancer cells were targeted using fucoidan/hyaluronic acid in order to reduce the resistance of the tumour to anticancer drugs. The therapeutic modality of the hybrid nanostructure was demonstrated by the ability of PG-carrying EGCG to suppress orthotopic prostate tumour growth.

Nanomaterials are not only used for synthesizing nanodrugs, but also as biosensors. Rauwel et al. [44] used confocal Raman spectroscopy to assess how easy it was for breast cancer cells and colorectal cancer cells to uptake cobalt metal nanoparticles. Raman spectra were acquired by means of a Nd:YAG laser at a wavelength of 532 nm. The apparatus was able to distinguish between the spectral signatures of extracellular and intracellular cobalt nanoparticles and could clearly show when the nanoparticles were localized inside the cancer cells. This permitted validating how easily the cobalt nanoparticles penetrated the membrane of the cancer cells, potentially causing cell apoptosis. 

Nanomedicine techniques are widely used in cancer diagnosis, screening, and therapy. In particular, cancer nanomedicine relies on a variety of spectroscopy techniques not only for characterizing anticancer drug nanocarriers or for imaging cancer tissues, but also for detecting cancer biomarkers and monitoring the effects of anticancer drugs on tumours. One of the most used spectroscopy techniques is Raman scattering because of its high flexibility and the high signal-to-noise ratio achieved by Raman scattering methods, such as Surface-Enhanced Raman Scattering (SERS), Tip-Enhanced Raman Spectroscopy (TERS), Coherent Anti-Stokes Raman Spectroscopy (CARS) [45], Resonance Raman Scattering (RRS), Surface-Enhanced Resonance Raman Scattering (SERRS), Spatially Offset Raman Spectroscopy (SORS), Transmission Raman Spectroscopy (TRS), and Stimulated Raman Scattering (SRS) [46]. Among these techniques, those which are largely employed in cancer diagnosis, staging, and therapy are spontaneous Raman scattering, SERS, SERRS, and SORS. CARS is mainly used for investigating the pharmacokinetics of anticancer drugs and nanocarriers both in vitro and in vivo, because of its high sensitivity and high-speed imaging [47]. Table 1 summarizes some of the cancer nanotherapies currently being investigated.

The present review focuses on the recent advancements in the application of spontaneous Raman scattering and different types of SERS to cancer nanotheranostics, the detection of extracellular vesicles as cancer biomarkers, and optical nanoprobes for cancer diagnostics, immunotherapy, and cancer bioimaging. 

The Raman techniques used to monitor the effects of anticancer drugs on cancer tissues are summarized in Table 2.

## 2. Spontaneous Raman Scattering

Raman spectroscopy techniques exploit the phenomenon of spontaneous Raman scattering, which was discovered by the Indian physicist Chandrasekhara Venkata Raman in 1927 [53], and for which he was awarded the Nobel Prize in 1930 [54]. Raman spectroscopy works by detecting the radiation inelastically scattered from a sample after irradiating it with a laser source, usually in the UV-Visible-Near IR range (~200 nm to ~800 nm wavelength). The photons from the incident laser light interact with the molecules of the sample, and the majority of the scattered photons have the same energy as the incident photons. Only a small portion of the photons are scattered at an energy that is either higher or lower than that of the incident photons, and it is this shift in energy (Raman shift) that gives us access to the vibrational energy difference from where the unique spectroscopic signature of the sample can be determined. 

Raman scattering methods are label-free and non-destructive and they are largely used for molecular cancer diagnostics because of their ability to detect changes in the biochemical signatures of cancer cells [55]. The success of Raman spectroscopy in nanomedicine lies not only in its ability to discriminate with high accuracy between healthy and diseased cells but also in its capability of identifying the unique biochemical fingerprints of individual cells and of tissues [56]. These fingerprints are generated by the vibrations of the molecular bonds of the components of the sample; in the case of cells and tissues, they are proteins, amino acids, nucleic acids, and lipids. Since the spectroscopic fingerprint region of cells and tissues ranges from about 400 cm^−1^ to about 2500 cm^−1^, Raman scattering techniques are suitable for investigating samples in physiological conditions and aqueous environments, allowing for both in vitro and in vivo applications. However, the long acquisition times required to collect just a handful of Raman photons (about 1 in 10^7^ photons) [57] do not facilitate the application of spontaneous Raman spectroscopy to clinical settings [58]. Despite that, spontaneous Raman scattering is widely exploited under laboratory conditions, and in the past decade, it has been extensively used for cancer diagnosis and cancer screening. 

### 2.1. Cancer Nanotheranostics

Theranostic nanomedicine is a nanotherapeutic system that integrates diagnostic imaging capability with therapeutic interventions [59]. In cancer nanomedicine, nanotheranostic was developed to counteract the challenges of effective cancer therapies caused by the molecular complexity of cancer. Since theranostics methods involve the use of molecular imaging tools, combinations of drug delivery systems with different imaging techniques (e.g., MRI, CT, or PET) were explored [60]. The theranostic combination in a single system of the benefits of the diagnostics and therapeutic capabilities of an agent makes nanoparticles an attractive cancer nanotheranostic system [61], with gold nanoparticles widely used as imaging contrast agents. This type of nanostructure is also employed in photothermal cancer therapy [62] because of the surface plasmon resonance, which seems to be linked to the localized irreversible cellular damage caused by irradiation, with light of a suitable wavelength, of the area where gold nanoparticles accumulate [63]. 

The fact that gold nanoparticles are employed in both Raman imaging and photothermal therapy has been used to implement cancer nanotheranostic approaches. Gao and co-workers [48] synthetized gold nanostars and assessed their suitability for Raman bioimaging (diagnostic) and tumour ablation (therapy) in a mouse model of orthotopic 4T1 breast cancer cells. Two gold nanostars with different extinction spectra were prepared by changing the HAuCl_4_·3H_2_O/HEPES (Au chloride trihydrate/N-(2-hydroxyethyl) piperazine-N′-(2-ethaneshlphonic acid)) ratio in solution: (1) Au nanostars-1 (10 mL of 140 mM HEPES mixed with 15 mL of 18 mΩ Milli-Q H_2_O and followed by the addition of 400 μL of 10 mg/mL HAuCl_4_·3H_2_O); and (2) Au nanostars-2 (22.5 mL of 140 mM HEPES mixed with 2.5 mL of 18 mΩ Milli-Q H_2_O and followed by the addition of 400 μL of 10 mg/mL HAuCl_4_·3H_2_O). The diagnostic capabilities of the nanotheranostic platform were explored through Raman bioimaging. 4T1 breast cancer cells were exposed to Au nanostars-1 and Raman images acquired by using a Horiba Jobin-Yvon Lab Ram HR-VIS high-resolution confocal Raman microscope with a laser source of 633 nm. The spectral images of the 4T1 breast cancer cells treated with Au nanostars-1 showed that after endocytosis the gold nanostars were located only in the cytoplasm. The therapeutic modality of the nanotheranostic system was tested by incubating Au nanostars-2 with 4T1 breast cancer cells in vitro. Exciting these nanostars with an 808 nm laser source, caused them to induce hypothermia in the cancer cells that led to the ablation of the tumour through photothermal therapy. 

The majority of theranostic systems employ nanoparticles; however, Raman scattering techniques have also been coupled with other diagnostic/therapy methods in order to create nanoparticle-free theranostic platforms. Horgan et al. [49] developed a nanoparticle-free cancer nanotheranostic system by combining Raman spectroscopy for cancer diagnosis with photodynamic therapy (PDT) as the therapeutic modality. They demonstrated that Raman microscopes with a laser source of 785 nm did not activate the photosensitisers used in PDT, hence showing the compatibility between the two techniques. The opposite was also true, with the photosensitiser 5-aminolevulinic acid (5-ALA)-induced protoporphyrin IX (PPIX) having the least effect on the Raman spectra of A549 lung carcinoma cells, MDA-MB-231 breast adenocarcinoma cells, and MDA-MB-436 breast adenocarcinoma cells. The validity of this nanoparticle-free cancer theranostic approach and its potential clinical application were demonstrated by carrying out an in vivo investigation of SW1222 colorectal tumour xenografts in *nu*/*nu* mice. The Raman spectra acquired from control mice and mice with the tumour xenografts before and 4 h after the administration of 5-ALA were comparable to the Raman spectra obtained in other in vivo studies. 

### 2.2. Monitoring of Anticancer Nanomedicine Therapeutics

Poojari et al. [50] investigated the potential of confocal Raman spectroscopy for assessing the response of tumoral tissues to anticancer nanodrugs. The spectral response of ex vivo hepatocellular carcinomas cells to two anticancer nano-agents — (1) microtubule-targeted vascular disrupting agents (MTVDA)-encapsulated non-targeted polymeric nanocomplexes PLGA-b-PEG-CA4 NP + PLGA-b-PEG-2ME NP combinatorial, and (2) MTVDA-encapsulated targeted cetuximab polymeric nanocomplexes Cet-PLGA-b-PEG-CA4 NP + Cet-PLGA-b-PEG-2ME NP combinatorial — were investigated by means of a confocal Raman microscope with a laser source of 532 nm. The Raman spectra of tumour tissues treated with anticancer drugs showed an increase in the intensity of the lipid peaks and of the amid-I bands. These two spectral features could be a clear indication of apoptotic activity in cancer cells as a result of the action of the anticancer agents. The Raman spectra of cohorts treated with different MTVDA encapsulated targeted cetuximab polymeric nanocomplex combinatorials showed clear spectral differences between the cohorts. The multivariate analysis made it possible to identify the Cet-PLGA-b-PEG-CA4 NP + Cet-PLGA-b-PEG-2ME NP combinatorial as that which achieved the highest stratification accuracy compared to the other combinatorials. These findings indicated Raman spectroscopy as a potential highly sensitive and rapid approach for therapeutic monitoring of anticancer nanodrugs on hepatocellular carcinomas. 

The potential of confocal Raman microspectroscopy as a label-free and non-destructive tool for molecular characterization of anticancer nanodrugs and their potential toxic effects on cells, was studied by Rammal and co-workers [51]. They investigated the activity of the anticancer chemotherapeutic drug doxorubicin within single murine lung carcinoma cells and human breast cancer cells. To facilitate the uptake of the anticancer drug from the cancer cells, doxorubicin was linked to the nanocarrier squalene because this bioconjugate spontaneously self-assembled in the form of nanoparticles when in water. The murine lung carcinoma cells and the human breast cancer cells were treated with 1 μM and 5 μM doxorubicin and squalene-doxorubicin bioconjugate (squalenoylated nanoparticles) and a confocal Raman microscope with an excitation source of 785 nm was used to locate the doxorubicin and squalenoylated nanoparticles in the nuclear region and in the cytoplasm of the murine lung and human breast tumours. The Raman spectra showed that after treating the murine lung carcinoma cells with 1 μM doxorubicin, the spectral signature of doxorubicin was detected neither in the nucleus nor in the cytoplasm of the cells. However, when the murine lung tumour cells were exposed to 1 μM squalenoylated nanoparticles, the Raman spectra displayed the peaks of the squalene-doxorubicin bioconjugate in both the nucleus and the cytoplasm of the cells. When the concentration of doxorubicin was increased to 5 μM and the murine lung cancer cells were exposed to it, not only were the Raman peaks of the anticancer drug observed in the nuclear region of the cells, but the intensity of the doxorubicin peaks at 1211 cm^−1^ and 1241 cm^−1^ also decreased, indicating intercalation of doxorubicin into DNA, which could account for the toxicity of the drug. Interestingly, when the cancer cells were exposed to 5 μM squaleoylated nanoparticles, the intensity of the Raman peaks of the squalene-doxorubicin bioconjugate increased in both the nucleus and the cytoplasm of the tumour cells, indicating an accumulation of the doxorubicin in both cellular regions. Additionally, the intensity of the 1211 cm^−1^/1241 cm^−1^ ratio was higher, which seemed to indicate a decrease in the intercalation of doxorubicin into DNA, which could lead to a decrease in the toxicity of the anticancer drug. 

### 2.3. Extracellular Vesicles (EVs) in Cancer Detection

The communication between cells and their environment occurs by means of different mechanisms involving

(1) Direct cell-to-cell contact;
(2) Secretions from cells of soluble factors, such as cytokines and growth factors;
(3) Extracellular vesicle (EV) trafficking.

EVs are secreted by many eukaryotic cells and can be found in bodily fluids, such as blood, urine, bile, and seminal and bronchoalveolar lavage fluids. EVs have different functions and sizes and are classified as

- Exosomes: they are <150 nm in diameter and are released to the extracellular environment after late endosomes have fused with the cell plasma membrane;
- Microvesicles and apoptotic bodies: they are >100 nm in diameter and are released from the cell plasma membrane into living (microvesicles) and dying (apoptotic bodies) cells [64].

EVs are important mediators of cell–cell communications because they are not only involved in many physiological processes (e.g., blood coagulation, stem cell differentiation, tissue regeneration, and embryo implantation), but also in pathological processes (e.g., neurodegenerative diseases and cancer). Despite the multiple challenges linked to the reliable and accurate detection of EVs, their involvement in many of the processes that contribute to the progression of cancer makes them ideal candidates as cancer biomarkers [65].

Krafft et al. [66] investigated the value of EVs (exosomes and microvesicles) as a diagnostic biomarker for tumour progression by comparing Raman spectra of cancer EV with those of non-cancer EV. Two different EV fractions (12,000× *g* for microvesicles followed by 120,000× *g* for exosomes) were isolated from the serum and plasma of healthy donors and cancer patients, and Raman spectra were acquired with an RXN1 Raman microscope equipped with a 785 nm laser source. The spectra showed that for cancer patients the exomes fraction contained more beta-sheet structure than the microvesicles fractions. These spectral differences were tested on EVs isolated from the serum and plasma of prostate cancer patients and benign prostatic hyperplasia patients, the latter acting as a control. Again, the Raman spectra showed an elevated content of beta-sheet proteins in prostate cancer EV as compared to benign prostatic hyperplasia EV. Since EVs play a key role in tumour growth and metastasis [67,68], these findings indicate that when EVs are secreted in the circulatory system and in the urinal tract, they can act as invaluable biomarkers for cancer detection and diagnosis.

Enciso-Martinez and co-workers [69] developed a new method for characterizing EVs from fluids based on trapping them and simultaneously acquiring their biochemical fingerprints. This was achieved by using a home-built synchronized Rayleigh and Raman scattering apparatus with a laser wavelength of 647.086 nm. The Rayleigh light scattering was detected when the EVs were trapped and Raman spectroscopy was employed to obtain the spectral signature of the trapped EV. This method was subsequently applied for distinguishing EVs derived from PC3 and LNCaP prostate cancer cells, from lipoproteins, and EVs originating from erythrocytes based on their Raman spectral signatures [70]. Comparison of the Raman spectra of tumour-derived EVs and lipoproteins showed that the peaks attributed to proteins (e.g., phenylalanine) were visible in the EVs’ spectra but not in the lipoproteins’ ones and that the lipid-protein peaks were more intense in the Raman spectra of EVs than in the spectra for lipoproteins. These findings seem to indicate that synchronized Rayleigh-Raman scattering may prove to be a promising label-free and non-invasive tool for the diagnosis of cancer. 

### 2.4. Raman Imaging

Raman imaging differs from Raman spectroscopy in that it makes it possible to obtain spectral and spatial information simultaneously. Raman images are chemical images in which each pixel contains spectral information because it is composed of a complete Raman spectrum. Raman images are acquired by mapping the sample area, namely by acquiring individual Raman spectra point by point within a defined area on the sample surface [71]. These images are called Raman hyperspectral maps where the step size of the mapping refers to the distance between two consecutive points within the chosen area and it provides the lateral resolution of the Raman map [72]. 

Bravo and co-workers [73] demonstrated the ability of spontaneous Raman bioimaging in screening and grading tumours at the cellular level. They used a Thermo Fisher Scientific DXR2xi Raman microscope with laser wavelengths 532 nm and 785 nm on osteo-differentiated mesenchymal stem cells (MSCs) and osteosarcoma cells. The Raman chemical maps obtained showed that the peaks for cysteine and hydroxyapatite at 668 cm^−1^ and 960 cm^−1^, respectively, were more intense in the Raman spectra of osteosarcoma cells than in those of osteo-differentiated cells. In addition, when Raman spectroscopy was used on chondrogenic tissues with different progressive tumorigenesis, it was able to give information on the grade of malignancy of the cartilaginous cancers. 

The exploration of spontaneous Raman bioimaging was continued by D’Acunto et al. [74] who employed a Raman imaging microscope with a laser wavelength of 532 nm to study the progression of enchondroma and chondrosarcomas and their grade of malignancy. Raman biochemical maps revealed a strong correlation between the progression of grading and the biochemical fingerprints of the extracellular matrix. In particular, the maps highlighted the relation between tumour grading and the alteration of proteoglycan contents in the biochemical makeup of non-collagenous proteins, as well as the progressive degradation of collagen type-II components. Additionally, Raman bioimaging showed that the Raman peaks assigned to proline and collagen appeared less intense going from enchondroma to chondrosarcoma grades 1 and 2, indicating proline as one of the biomarkers of the collagen degradation that occurs in tumours. Chondrosarcoma grade 3 behaved differently as it displayed Raman spectra in which the peaks assigned to tyrosine, tryptophan, and phenylalanine intensified. Multivariate analysis of the Raman maps showed that Raman spectroscopy was able to distinguish between the chondrogenic tumours grades 1 and 2 with a 90% sensitivity, 90% specificity, and 90% accuracy, and between the enchondroma and chondrosarcomas with ca. 75% sensitivity, 75% specificity and 75% accuracy, which clearly show the potentially significant impact that Raman spectroscopy can have in the diagnosis and progression of grading of cartilaginous tumours. Table 3 summarizes the cancer Raman imaging techniques currently used. 

## 3. Surface-Enhanced Raman Scattering (SERS)

In 1973, Fleischmann and co-workers [80] carried out Raman spectroscopy of pyridine adsorbed at a silver electrode that led to the development of surface-enhanced Raman scattering (SERS) [81], which combines the high flexibility and specificity of spontaneous Raman spectroscopy with the increased sensitivity caused by the signal amplification generated through the use of plasmonic nanostructures. The surface plasmon resonance generated by the collective oscillations of the electrons in the electronic conduction bands of plasmonic nanoparticles causes an increase in the intensity of the Raman spectra [82], which makes SERS an attractive technique in cancer diagnosis and therapy [83]. Table 4 summarizes some of the current Raman-based cancer diagnosis procedures. Due to its high sensitivity, specificity and accuracy, SERS is not only used for the detection of cancer but also for staging through the chemometric analysis of cancer biomarkers (e.g., cellular metabolites and exosomes) that circulate in extracellular bodily fluids, such as blood and urine [84]. 

SERS is also used for investigating and quantifying the profiles of the release of anticancer drugs. Managò et al. [52] designed a nanocarrier for the delivery of the molecule Galunisertib (LY2157299, LY) to colorectal cancer cells (CRCs). LY presents anticancer properties because of its ability to block the transforming growth factor-β1 receptor, which is responsible for the epithelial-to-mesenchymal transition that allows CRCs to migrate and metastasize. The nanocarrier was constituted by a diatomite nanoparticle decorated with gold nanoparticles and enclosed in a gelatin shell to retain LY and deliver it to CRCs. The amount of LY released to living CRCs, and the subsequent increase in the ability of the anticancer drug to reduce cancer migration and metastasis, were quantified by SERS imaging of LS-174T colorectal cancer cell line after cellular uptake of the nanocarrier. SERS spectra and images were acquired with an Xplora Inv. Horiba–Jobin Yvon confocal Raman microscope with a laser source of wavelength 638 nm. These results showed the potential, using this hybrid nanovector, for the delivery of a minimal amount of anticancer drug to cancer cells to decrease the number of toxic metabolites that are released with the drug. 

### 3.1. SERS Nanoprobes for Cancer Diagnosis

SERS nanoparticles are the molecular contrast agents and nanobiosensors [85] used in in vivo cancer Raman imaging and in vitro Raman spectroscopy. These nanoparticles have many advantages when it comes to clinical translation; for example:

(1) The materials used to make SERS nanoparticles are inert;
(2) Passive or non-targeted (i.e., without any targeting moiety) resonant SERS (SERRS) nanoparticles (NPs) can be synthesized. SERRS NPS are not only capable of visualizing different types of cancer, but they also make it possible to image local metastases in animal models;
(3) Active or targeted (i.e., with a targeting moiety) SERRS NPs make it possible to visualize in vivo tumoral extensions that otherwise would go unobserved;
(4) SERS multiplexing can be achieved where SERS nanoparticles of the same size and chemical composition can be spectrally different from one another, thus allowing multiplex detection of different types of cancer [86].

SERS analysis for cancer diagnosis can be

- Direct/label-free;
- Indirect/encoded SERS.

#### 3.1.1. Label-Free SERS

In direct SERS, plasmonic nanoparticles are used as SERS substrates by adsorbing cancer biomarkers on their surfaces. Karunakran et al. [87] used label-free SERS for accurate differential grading of normal cervical cells (control), high-grade squamous intraepithelial lesions (HSIL), and cervical squamous cell carcinoma (CSCC), through the SERS-aided grading of cervical exfoliated cells obtained from cervical smear tests. Gold nanoparticles were used as SERS substrates and incubated with cervical exfoliated single cells. SERS spectra were acquired using a WITec alpha 300 R confocal Raman microscope equipped with a 633 nm laser source and showed the presence of distinctive Raman peaks for the three grades. In particular, high nuclear content and high protein content appeared to be observed in HSIL and CSCC compared to normal cervical cells. A shift in the amide III signal from proteins from 1262 cm^−1^ in normal cells to 1270 cm^−1^ in HSIL and CSCC was observed along with a shift of 10 nm of the amide II peak at 1560 cm^−1^ from normal cells to HSIL and CSCC. Cervical exfoliated cell pellets were also studied; since they contained a mixture of normal cervical cells, HSIL, and CSCC, they showed Raman features common to the three grades. Finally, the nucleic acids profiling was rechecked by acquiring the SERS spectra of the DNA extracted from cervical exfoliated cells. As expected, the majority of the Raman peaks in the SERS spectra from the extracted DNA correlated with those in the SERS spectra obtained from cervical exfoliated single cells and pellets. The findings indicated that a SERS spectro-cytology approach based on the use of exfoliated cells can lead to a more sensitive grading of cervical precancerous lesions and cervical carcinomas. 

#### 3.1.2. Encoded SERS

SERS-encoded nanoparticles (SEPs) are hybrid nanostructures with a plasmonic nanoparticle core and coated with a SERS reporter on which a protective layer of silica is often added [88]. SEPs come in different shapes (sphere, star, and rod) depending on how intense the SERS signal is to be. Of the three shapes, SERS nanorods appear to be the best option because not only are the SERS intensities higher than those of SERS spheres due to the accumulation of the electromagnetic field to their tips, but the geometry of these nanoparticles can be precisely controlled so that a homogenous SERS signal can be achieved. SERS nanostars also produce higher intensities than spheres, but contrary to the nanorods, the geometry of the nanostars’ tips are inhomogeneous, leading to great variance in the SERS intensities of different nanostars [89]. 

#### 3.1.3. SERS Characterization of EVs

Direct and indirect label-free SERS analyses of EVs are used for cancer diagnosis. At this end, the chemico-physical properties of EVs are exploited to make the vesicles adhere to plasmonic nanostructures in order to detect the SERS signal of EVs. However, direct SERS analysis poses many challenges due to the small sizes of exosomes and their complex compositions. In fact, exosomes contain biomolecules whose Raman signal is intrinsically weak, leading to an overall Raman spectrum showing broad peaks that make it difficult to discriminate the spectral fingerprint of EVs. When exosomes are allowed to directly interact with plasmonic nanostructures in order to increase the intensity of the EVs SERS signal, one of the main difficulties is to ensure that the nanostructures are regularly arranged on the exosome outer layer. To overcome these obstacles, the SERS spectra of exosomes can be analysed using deep learning algorithms in order to distinguish between cancer and healthy patients [90]. 

A more promising approach to the detection of exosomes is the indirect SERS analysis, which exploits the conjugation of SEPs with targets in order to ensure the accumulation of SEPs on exosome surfaces. Capturing substrates (e.g., magnetic beads) are also used to make sure that the SEPs-decorated exosomes accumulate but they are still sufficiently separated in small areas in order to increase the sensitivity of the exosome detection [91].

Osei et al. [92] used SERS to characterize the spectral signatures of EV-enriched fractions from the plasma of prostate cancer patients. The EV fractions were incubated with silver nanoparticles and SERS spectra were acquired by means of a Raman spectrometer RXN1 with a laser source of 785 nm. The spectra for cancer samples displayed an increase in the intensity of the Raman peaks for proteins at 382, 394, 713, 854, 1004, 1132, 1238, and 1393 cm^−1^ compared to spectra for control samples from non-cancer (control) patients. These bands could be considered as potential prostate cancer biomarkers. Furthermore, differences between the Raman bands for disulfide stretches and tryptophan residues in proteins in the SERS spectra of EV fractions from control and cancer patients were observed. These preliminary results seem to show the potential of SERS analysis of EV-enriched fractions to discriminate between cancer and non-cancer patients. 

#### 3.1.4. Multiplexing

SEPs are particularly useful when used as contrast agents for multiplex analysis, characterization of individual cancer cells and tumoral tissues, and cancer diagnosis by means of in vitro and in vivo bioimaging. In particular, multiplexing has proved to be an invaluable technique in cancer molecular biology, immunohistochemistry, and diagnosis because it allows the quantification of multiple receptors in parallel using just one sample. Li et al. [93] used multiplexing SERS to achieve ultrasensitive detection of multiple soluble cancer protein biomarkers—for example, soluble programmed death 1 (sPD-1), soluble programmed death-ligand 1 (sPD-L1), and soluble epithermal growth factor receptor (sEGFR)—which are linked to the progression of the tumour, as well as the efficacy of the cancer therapy. The SERS nanoprobes were synthetized using anisotropic Au–Ag alloy nanoboxes as SERS substrates and specific nanoyeast-single-chain variable fragments (nanoyeast-scFvs) as affinity reagents. The nanoyeast-scFvs were obtained by embedding single-chain variable fragments (scFvs) in fragments of yeast cell walls. The SERS signals were recorded using a portable IM-52 Raman microscope with a laser source of 785 nm. The cancer biomarkers were successfully detected with limits of detection of 6.17 pg/mL for sPD-1, 0.68 pg/mL for sPD-L1, and 69.86 pg/mL for sEGFR, demonstrating the potential of multiplexed SERS detection of soluble cancer protein biomarkers as a cancer diagnostic tool. 

#### 3.1.5. SERS Immunoassay and Immunotherapy

SERS immunoassays are proving to be highly successful in the detection of cancer biomarkers and show great potential as cancer diagnosis tools in clinical settings. In particular, some cancer immunotherapies use SERS-active nanoparticles as immunomarkers for immunotherapy stratification. SERS-active nanomaterials are also employed for immunomarker biosensing where antibody-coated plasmonic SERS nanoparticles are incubated with target immunomarkers. SERS images are subsequently acquired, which clearly show the distribution of target biomarkers on the cancer cell surface [94]. 

Koo et al. [95] used SERS as a nanodiagnostic tool for early prostate cancer risk stratification by integrating it with Mi-Prostate Score (MiPS), a clinically validated prostate cancer risk score test that, however, is currently carried out through a lengthy laboratory process. MiPS is a urine test for detecting two prostate cancer biomarkers: a piece of RNA made from the prostate cancer antigen 3 (PCA3), and another RNA marker that is found only when TMPRSS2 and ERG abnormally fuse (T2:ERG) [96]. The SERS signals for the RNA biomarkers T2:ERG and PCA3 in cancer patient urine samples were obtained by adsorbing T2:ERG and PCA3 amplicons onto 40 nm cationic silver nanoparticles. The algorithm of MiPS was then followed and the cancer biomarker SERS signals were used to develop a prostate cancer risk score. Comparison of the %SERS_MiPS score with patients’ biopsy outcomes led to a clinical sensitivity of the SERS_MiPS of 87% and a specificity of 90%, which confirmed the potential of this new cancer-nanodiagnostic tool to accurately separate patients into different risk groups. 

SERS immunoassay platforms can also be used for the high sensitivity analysis of cancer biomarkers in human serum. Wang and co-workers [97] employed a double antibody sandwich-type SERS immunoassay for detecting Interleukin 8 (IL-8) in serum because of its fundamental role in tumour growth and angiogenesis and, therefore, its importance in cancer early diagnosis as well as in cancer treatment. The sandwich was formed by gold nanocages (GNCs) modified with IL-8 that acted as SERS tags, and by highly branched gold nanoparticle substrates (HGNPs) conjugated with IL-8 that were employed as SERS capturing substrates. The IL-8 SERS tags were fabricated using 4-mercaptobenzoic acid (4-MBA) as both a Raman reporter and a covalent link to the anti-IL-8. The antigen-IL-8-conjugated HGNP capturing substrates were obtained by placing a piece of ITO glass functionalized with (3-Aminopropyl)triethoxysilane (APTES) in an HGNP colloidal solution so that when the pendant primary amine groups of APTES were covalently bound to the nanoparticles, the HGNP substrate was formed. Combining the IL-8 antigen and antibody enabled the construction of a SERS immunosensor for the detection of IL-8. The SERS measurements were carried out using a Renishaw Raman system-in-Via-Reflex equipped with a 785 nm laser. The Raman spectra of serum from gastric cancer and breast cancer patients showed that the intensity of the peak at 1077 cm^−1^ was six to eight times higher than in the spectra of the serum from healthy subjects, indicating a higher concentration of IL-8 in cancer patients compared to healthy subjects. 

### 3.2. SERS Bioimaging

One of the main applications of SERS is in cancer imaging due to the flexibility that using SERS nanoparticles as contrast agents and nanosensors allows. In particular, new efficient and safe-to-use SERS nanoparticles have been developed and synthetized for in vivo imaging [98]. 

SERRS NPs have been found to be very promising molecular contrast agents for in vivo tumour imaging because altering the way they are synthetized leads to the shifting of their limits of detection by orders of magnitude. Additionally, the high Raman signal specificity and sensitivity obtained in cancer Raman imaging when using SERRS NPs as contrast agents not only makes it possible to achieve universal cancer imaging because SERRS NPs can overcome the barrier posed by the EPR effect, but it also permits the clear visualization of the delineation of the cancer tissue [99]. Oseledchyk et al. [75] used folate receptor (FR)-targeted SERRS NPs as a contrast agent for ovarian cancer imaging because folate receptors are overexpressed in ovarian cancer cells. They synthetized two different SERRS NPs: (1) a targeted nanoprobe functionalized with an anti-folate-receptor antibody via a PEG-maleimide-succinimide crosslinker with infrared dye IR780 as the Raman reporter; and (2) a non-targeted nanoprobe coated with PEG_5000_-maleimide with infrared dye IR140 as the Raman reporter. A Topically Applied Surface Enhanced Resonance Raman Ratiometric Spectroscopy (TAS3RS) method was developed, which used an inVia RFenishaw Raman microscope equipped with a 785 nm diode laser. TAS3RS was based on the ratiometric information obtained from the difference between anti-FR-SERRS nanoparticle homing and non-targeted SERRS nanoparticle homing. Tumour lesions of any size (down to a few hundred μm) and at any location in murine animal models of human ovarian adenocarcinoma were successfully detected, demonstrating the great potential of TAS3RS for the intraoperative detection of microscopic residual cancer tissues. 

SERRS techniques also hold great potential for visualising the true extension of brain tumours. Huang and co-workers [76] synthetized integrin-target SERRS nanoparticles to visualize the true extent of a glioblastoma multiforme (GBM) tumour in an animal model. They used Raman multiplexing to compare the RGD-peptide-conjugated version for integrin-targeting (RGD-SEERS) to the non-targeted RAD-SERRS control in the same animal models. Multiplexed Raman images of mice bearing GBM cancers were acquired with a Renishaw InVia Raman microscope equipped with a 785 nm diode laser. The non-targeted RAD-SERRS nanoparticles were able to visualize the delineation of the main GBM tumoral tissue; however, integrin-targeting RGD-SERRS nanoparticles had the ability to clearly image the real extension of the main tumoral tissue including its margins. 

SERRS NPs hold great potential in Raman cancer imaging and it is possible to synthetize them with limits of detection below the femtomolar region (≤10^−15^ M) [100]. However, this technology has limitations in a clinical setup because SERRS NPs are not commercially available and Raman imaging systems are not widely used in clinical settings. Furthermore, the protocols for synthetizing SERRS NPs are optimized for a limited range of wavelengths, mainly in the infrared region, which limits the depth of tissue penetration to millimetres. To overcome this limitation and increase the detection of SERRS NPs into the tissue to the centimetres depth, surface-enhanced spatially offset Raman scattering (SESORS) can be used, where spatially offset Raman spectroscopy (SORS) is employed to detect SERRS NPs [101].

The benefits and the capability of SESORRS for non-destructive in vivo imaging of different types of cancer were shown by Nicolson et al. [77] who used SESORS for the in vivo visualization of deep-seated glioblastoma multiforme (GBM) tumours through the intact skull of mice. Integrin-targeting SERRS active nanostars were synthetized, which could target only GBM tumours. In vivo SORS imaging of GBM cancer-bearing mice was carried out by using a custom-built SORS system equipped with a 785 nm laser source. The surface-enhanced spatially offset resonant Raman scattering (SESORRS) technique was able to acquire clear images of the extension of the deep-seated GBM non-invasively. The SESORRS images were not only comparable to those obtained using MRI, but they also displayed high contrast and high signal specificity for the tumour region. 

#### Immuno-SERS (iSERS) Microscopy

The advantage of immuno-SERS (iSERS) microscopy on standard Raman microscopy is speed. The acquisition times per pixel of iSERS microscopy are more than one order of magnitude lower than the times achieved by the first iSERS microscope in 2006 [102], due to the use of high laser power densities as laser sources. In particular, Wang et al. [103] demonstrated that using 1–2 mW laser power with a 50 ms acquisition time per pixel led to the collection of highly reproducible iSERS images in repeated experiments performed on the same individual breast cancer cell overexpressing the biomarker human epidermal growth factor receptor 2 (HER2). 

Stepula et al. [78] further developed iSERS microscopy and designed a multicolour/multitarget SERS imaging technique that would allow the characterization of the expression of different cancer protein biomarkers on the same single cancer cell. In particular, they developed a 6-colour/1-target iSERS microscope and successfully localized HER2 on single SkBr-3 breast cancer cells. This was achieved by labelling the corresponding antibody (anti-HER2) with six different small (<80 nm) SERS nanotags (i.e., Au/Au core/satellite particles with six distinct Raman reporter molecules on the Au core) with single-particle brightness. The iSERS mapping was carried out using a WITec alpha 300 R Raman imaging microscope with only one laser source of 632.8 nm wavelength and 1.2 mW power. The spectra of the six SERS nanotags could be clearly distinguished on the Raman maps of one single SkBr-3 cell. These results demonstrate the great potential of multicolour iSERS mapping on one single cell for the identification of unknown protein markers expressed on the surface of cancer cells that could further enable the early detection of cancer. 

Stepula and co-workers [79] also used Au/Au core/satellite nanoparticles with Raman reporter molecules on the surface as SERS nanotags to obtain false-colour iSERS images of the specific location of programmed cell death-ligand 1 (PD-L1) on a single SkBr-3 breast cancer cell. The SERS nanotags were bioconjugated to antibodies (anti-PD-L1) and the SkBr-3 cell slides were first incubated with the primary antibody and, after that, the slides were stained with the SERS-labelled secondary antibody (positive control). Negative control slides were also prepared where the SERS-labelled secondary antibody was incubated on the SkBr-3 cell slides but without first incubating the slides with the primary antibody. The false-colour iSERS images of the positive controls clearly showed the selective localization of PD-L1 on the cell membrane of the cancer cells. 

## 4. Conclusions

Raman spectroscopy, in its various forms, has proved to be a highly flexible, accurate, efficacious, and multiplexing method in cancer nanomedicine. Its non-destructive and label-free approach to sample characterization, along with its high specificity and sensitivity in probing the biochemical fingerprints of individual cells and tissues, have contributed to its current widespread use in cancer diagnostics and therapy. One of the areas where Raman scattering techniques are being developed very rapidly is Raman bioimaging for diagnostic purposes. In particular, new developments in spontaneous Raman and surface-enhanced Raman spectroscopy (SERS) techniques to further improve the speed of data acquisition, as well as the specificity and sensitivity for in vitro and in vivo experiments, are instrumental in promoting the application of Raman spectroscopy and imaging to clinical settings. Despite the fact that, in the past few years, Raman bioimaging has achieved high levels of resolution and sensitivity due to the use of SERS nanoparticles and Resonant SERS (SERRS) nanoparticles as contrast agents, the clinical application of bioimaging to cancer diagnosis is still very limited. This is primarily because most SERS microscopes are unable to penetrate deep tissues. To overcome this limitation, bioimaging systems based on surface-enhanced spatially offset Raman scattering (SESORS) have been developed, and have shown great potential for imaging deep-seated tumours. Another challenge for the clinical use of Raman imaging lies in achieving a fast enough acquisition speed. To address this issue, immuno-SERS (iSERS) microscopy has been developed, which has proven to be sufficiently fast and able to produce reliable Raman images for the characterization and localization of tumour biomarkers on the surface of cancer cells.

Raman spectroscopy is also used in theranostics. Recently, new Raman-based cancer nanotheranostic platforms have been developed, where plasmonic nanoparticles are employed to enhance the Raman signal from the cancer cells and obtain high quality Raman maps, as well as to act as therapeutic anticancer agents. To overcome the toxicity issues that might be related to the use of nanostructures, nanoparticle-free theranostic systems have also been investigated where Raman bioimaging (diagnostic modality) is coupled with an anticancer therapy, such as photodynamic therapy. 

Raman spectroscopy methods have also been employed for the detection of cancer via unique biomarkers. Extracellular vesicles, such as exosomes, are excellent candidates as cancer biomarkers because of their being involved in many of the processes that contribute to tumour growth. In particular, confocal Raman spectroscopy and SERS have been employed to discriminate EV-enriched fractions from the plasma and serum of cancer patients. Detection of cancer biomarkers has also been successfully achieved by using SERS-based cancer immunotherapy, indicating its great potential for clinical cancer diagnostics.

Although recent years have witnessed a rapid development in Raman spectroscopy and imaging techniques that have led to some clinical applications of Raman-based systems, there are still factors (e.g., optimal excitation laser wavelength) that limit the promotion of Raman scattering techniques in clinical settings. In addition, the development of portable and commercial Raman devices with clinical capabilities and modalities for cancer diagnostics and therapy is a challenge that holds great potential for the future of Raman spectroscopy in clinical cancer nanomedicine, but that still requires further research.

## Figures and Tables

**Table 1:** Summary of some of Raman based cancer nanotherapies currently being investigated.

Therapy Type	Modality	Nanoparticles	Anticancer Drug	Raman Instrumentation	Target	Reference
NP assisted theranostic platform	Diagnostic in vivo molecular imaging	FU/HA/PG- Coated- PLGA- Encapsulated Iron oxide	Epigallocatechin gallate (EGCG)	T2-weighed MRI and in vivo imaging system-CT	Orthotopic mouse model of prostate cancer	[43]
	Therapeutic Activated Nanoparticle anticancer drug delivery	FU/HA/PG- Coated- PLGA		Fluorescence microscopy and Confocal spontaneous Raman Microscopy (excitation at 488 nm and emission at 525 nm)		
NP assisted theranostic platform	Diagnostic Raman bioimaging	Au nanostars-1	No	Confocal Spontaneous Raman Microscopy (λ = 633 nm)	Orthotopic mouse model of breast cancer	[48]
	Therapeutic tumour ablation	Au nanostars-2	No	Photothermal Therapy (λ = 808 nm; density = 1 W/cm^2^; thermographs recorded with a NIR camera at intervals of 5 s)		
NP-free theranostic platform	Diagnostic Raman bioimaging	No	No	Spontaneous Raman Microscopy (λ = 785 nm; laser power on sample ~80 mW; integration time = 10 s; number of spectra acquired for each cell = 5)	Colorectal tumour xenografts in *nu*/*nu* mice	[49]
	Therapeutic Photodynamic therapy	No	No	Photosensitiser 5-ALA and laser source (λ = 785 nm)		

**Table 2:** Summary of some of the Raman techniques used for monitoring the effects of anticancer drugs on cancer tissues.

Monitoring Approach	Nanoparticles	Anticancer Drug	Raman Instrumentation	Target	Reference
Cancer response to anticancer drugs	Targeted Cet-PLGA- b-PEG and Non-targeted PLGA- b-PEG	Microtubule targeted vascular disrupting agents (MTVDA)	Confocal Spontaneous Raman Microscopy (λ = 532 nm; laser power on sample = 10 mW; integration time = 5 s; number of spectra acquired for each sample = 10)	Ex-vivo hepatocellular carcinomas	[50]
Monitoring of concentration of anticancer drug in cancer cell nucleus/ cytoplasm	Squalene	Doxorubicin	Confocal Spontaneous Raman Microscopy (λ = 785 nm; laser power on sample = 60 mW; integration time = 20 s; number of spectra acquired for each sample = 30)	Murine lung carcinomas and Human breast cancer	[51]
Assessing profiles of the release of anticancer drugs	Diatomic NP decorated with Au NPs and enclosed in gelatin shell	Galunisertib	SERS (λ = 638 nm; He-Ne laser power = 50 mW; laser power on sample = 1 mW; acquisition time = 1 s; SERS spectra collected from 30 cells) Raman imaging (λ = 638 nm; He-Ne laser power = 50 mW; laser power on sample = 20 mW; Raman images acquired by raster scanning with step size of 0.5 μm; number of spectra acquired per cell = 1500/2000)	Colorectal cancer	[52]

**Table 3:** Summary of current cancer Raman bioimaging techniques.

Raman Imaging	Application	Nanoparticles	Raman Instrument	Target	Reference
Spontaneous Raman microscopy	Cancer screening and grading	No	Raman microscope (λ = 785 nm; power on sample = 5–10 mW; integration time = 1 s; 10 scans per spectrum)	Osteosarcoma	[73]
Spontaneous Raman microscopy	Cancer progression and grading	No	Raman microscope (λ = 532 nm; power on sample = 5–10 mW; integration time = 1 s; 10 scans per spectrum)	Enchodroma and Chondrosarcoma	[74]
Topically Applied Surface Enhanced Resonance Raman Ratiometric Spectroscopy (TAS3RS)	Visualization of microscopic residual of ovarian cancer tissues	Targeted functionalized NPs with IR780 Raman reporter and Non-targeted NPs with IR140 Raman reporter	Raman microscope (λ = 785 nm; diode laser power = 300 mW; power on sample = 100 mW; acquisition time < 100 ms)	Murine animal model of ovarian adenocarcinoma	[75]
Raman multiplexing	Visualization of true extension of brain cancer	Targeted-RGD-SEERS and Non-targeted RAD-SEERS	SERS (λ = 785 nm; diode laser power = 300 mW; power on sample = 10–100 mW; acquisition time = 1.5 s)	Glioblastoma multiforme (GBM) Cancer	[76]
SESORRS	In-vivo visualization of cancer tissues	Integrin-targeting SERRS active nanostars	SORS (λ = 785 nm; laser power = 400 mW; power on sample for conventional Raman imaging = 20 mW; power on sample for SORS measurements = 130 mW)	Deep-seated GBM tumour through intact skull	[77]
Multicolour/multitarget SERS imaging	Characterisation of expression of different cancer protein biomarkers on the same cancer cell	SERS Au/Au core satellite NPs with 6 distinct Raman reporters on the core	6-colour/ 1-target iSERS microscope (λ = 785 nm; He-Ne laser power on sample = 1.2 mW; integration time = 100 ms)	Breast cancer	[78,79]

**Table 4:** Summary of current Raman approaches to cancer diagnosis.

Diagnosis Type	Nanoprobes	Raman Instrument	Target	References
Cancer biomarkers	EVs-fractions from serum and plasma of cancer patients	Spontaneous Raman microscopy (λ = 785 nm)	Prostate cancer	[66]
Cancer biomarkers	Cancer-derived EVs	Synchronized Rayleigh- Spontaneous Raman Apparatus (λ = 647.089 nm; laser power on sample = 70 mW; number of spectra acquired = 256; acquisition time per spectrum = 38 ms over a period of ~9.7 s; number of spectra per measurement = 25,600)	Prostate cancer	[69]
Differential grading	Au NPs	SERS (λ = 633 nm; laser power on sample = 3–7 mW; Stokes shift Raman spectra: integration time = 10 s; number of accumulations = 10 Single cell Raman mapping: map size = 5 μm × 5 μm; number of points per line = 150 × 150; integration time per point = 0.02 s)	High-grade squamous intraepithelial lesion (HSIL) and cervical squamous cell carcinoma (CSCC)	[87]
Cancer biomarkers	EVs incubated with Ag-NPs	SERS (λ = 785 nm; laser power = 150 mW; SERS spectra of EV-enriched Ag-NPs suspensions: acquisition time = 10 s; number of SERS spectra acquired/droplet = 5 Raman maps of dried film of EV-enriched deposited on CaF_2_ substrate: Exposure time = 10 s; step size = 10 μm)	Prostate cancer	[92]
Multiplex SERS for cancer detection	Au-Ag-alloy nanoboxes	SERS (λ = 785 nm; laser power = 70 mW)	Soluble cancer protein biomarkers	[93]
Cancer risk stratification	Cationic Ag-NPs	MiPS and SERS (λ = 785 nm; laser power = 70 mW; acquisition time = 1 s; number of measurements/sampled volume = 10)	Prostate cancer	[95]
SERS immunosensor for detection of cancer biomarker IL-8	Antibody sandwich SERS made of Au-nanocages and highly branched Au-NP substrate	SERS (λ = 633 nm; He-Ne laser power = 5 mW; acquisition time = 10 s; number of serum samples = 30)	Gastric cancer and breast cancer	[97]

## Data Availability

Not applicable.

## Abbreviations

ALA	Amino-Levulinic Acid
APTES	(3-Amino-Propyl)Tri-Ethoxy-Silane
CA4	Cyproterone Acetate
CARS	Coherent Anti-Stokes Raman Spectroscopy
Cet	Cetuximab
CRCs	Colo-Rectal Cancer cells
CSCC	Cervical Squamous Cell Carcinoma
CT	Computed Tomography
DNA	Deoxyribo-Nucleic Acid
EGCG	Epi-Gallo-Catechin Gallate
EPR	Enhanced Permeability and Retention
ERG	ETS transcription factor
ETS	E26 Transformation-Specific
EVs	Extracellular Vesicles
FR	Folate Receptor
GBM	Glio-Blastoma Multiforme
GNCs	Gold Nano-Cages
HER2	Human Epidermal growth factor Receptor 2
HGNPs	Highly branched Gold Nanoparticles
HSIL	High grade Squamous Intraepithelial Lesion
IL-8	Interleukin 8
iSERS	Immuno-Surface-Enhanced Raman Spectroscopy
IR	Infra-Red
4-MBA	4-Mercapto-Benzoic Acid
ME	Methyl Group
MiPS	Mi-Prostate Score
MRI	Magnetic Resonance Imaging
MSCs	Mesenchymal Stem Cells
MTVDA	Microtubule-Targeted Vascular Disrupting Agents
Nd:YAG	Neodymium doped Yttrium Aluminium Garnet
NPs	Nanoparticles
PCA3	Prostate Cancer Antigen 3
PDT	Photo-Dynamic Therapy
PEG	Poly-Ethylene Glycol
PET	Positron Emission Tomography
PLGA	Poly (D,L-Lactide-co-Glycolide)
PPIX	Proto-Porphyrin IX
RGD	Arginylglycylaspartic Acid
RRS	Resonance Raman Scattering
scFvs	Single Chain Variable Fragments
sEGFR	Soluble Epithermal Growth Factor Receptor
SEPs	SERS Encoded Nanoparticles
SERS	Surface-Enhanced Raman Spectroscopy
SERRS	Surface Enhanced Resonance Raman Scattering
SORS	Spatially Offset Raman Spectroscopy
sPD-1	Soluble Programmed Death 1
sPD-L1	Soluble Programmed Death-Ligand 1
SRS	Stimulated Raman Scattering
TAS3RS	Topically Applied Surface Enhanced Resonance Raman Ratiometric Spectroscopy
TERS	Tip Enhanced Raman Spectroscopy
TMPRSS2	Transmembrane Serine Protease
TRS	Transmission Raman Spectroscopy
UV	Ultra-Violet
